# Preoperative CT diagnosis of aberrant right subclavian artery and prediction of a nonrecurrent laryngeal nerve: a case report

**DOI:** 10.1097/RC9.0000000000000894

**Published:** 2026-08-21

**Authors:** Shuxiong Li, Zhanhui Chen, Yunqing Li, Dongwei Li

**Affiliations:** a Department of General Surgery, Section 1, Dongguan Tungwah Hospital, Dongguan, Guangdong, China; b Department of The First Clinical Medical College, Guangdong Medical University, Zhanjiang, Guangdong, China; c Reproductive Medicine Department, Affiliated Hospital of Guangdong Medical University, Zhanjiang, Guangdong, China

**Keywords:** aberrant right subclavian artery, case report, computed tomography, nerve protection, non-recurrent laryngeal nerve, thyroid surgery

## Abstract

**Introduction:**

The nonrecurrent laryngeal nerve (NRLN) is a rare anomaly associated with an aberrant right subclavian artery (ARSA). Preoperative recognition is critical to prevent injury during thyroid surgery.

**Case presentation:**

A 24-year-old male presented with a right neck mass. Preoperative contrast-enhanced CT revealed ARSA originating from the aortic arch and passing behind the esophagus, suggesting a right NRLN. During endoscopic bilateral total thyroidectomy via a breast approach (ETBA), a Type I NRLN (Toniato) was identified originating directly from the cervical vagus and entering the larynx laterally; it was preserved intact. The patient recovered uneventfully, with normal vocal function at discharge and throughout 14 months of follow-up.

**Discussion:**

CT detection of an ARSA should alert surgeons to the possibility of an NRLN, guiding intraoperative dissection away from the tracheoesophageal groove toward the vagal trunk.

**Conclusion:**

Preoperative CT is a valuable tool for predicting NRLN. Surgeons should remain vigilant when ARSA is present to enable early nerve identification and preservation.

## Introduction

The non-recurrent laryngeal nerve (NRLN) is a rare anatomical variation of the recurrent laryngeal nerve (RLN), first identified and described by George William Stedman in 1823^[^1^]^. The lesion originates from the abnormal development of the fourth pair of arch arteries during the embryonic period, leading to vascular malformation and subsequent failure of the recurrent laryngeal nerve to return along its normal pathway^[^2^]^. The incidence of right NRLN is approximately 0.3-0.8%, whereas left NRLN is even rarer, accounting for only about 0.004%^[^3^]^.

During thyroid surgery, accidental injury to the NRLN may lead to severe complications, including permanent hoarseness, aspiration, and airway obstruction. Under normal circumstances, the RLN passes around the right subclavian artery or the left aortic arch, then follows a predictable path along the tracheoesophageal groove^[^4^]^. Unlike the RLN, the NRLN can extend directly and horizontally to the larynx from the vagus nerve. This anomalous course renders it highly susceptible to injury during thyroidectomy at the superior pole or during ligation of the superior thyroid artery.

HIGHLIGHTS

- The right NRLN predicted by preoperative CT in this case was confirmed during surgery and identified as Toniato Type I.
- Despite the absence of IONM, endoscopic BABA thyroidectomy achieved safe nerve preservation.
- Preoperative CT identification of ARSA alerts surgeons to NRLN, guiding dissection toward the cervical vagal trunk and away from the tracheoesophageal groove, thereby reducing the risk of nerve injury.
- The patient maintained normal vocal function over a 14-month follow-up.

Preoperative imaging studies, particularly contrast-enhanced CT, are crucial for detecting ARSA and predicting the presence of NRLN. This case report presents a preoperative CT scan that identified ARSA and successfully predicted NRLN, thereby enabling intraoperative recognition and protection.

This case report has been reported in line with the SCARE 2025 criteria^[^5^]^.

## Clinical case

**Figure 1.**
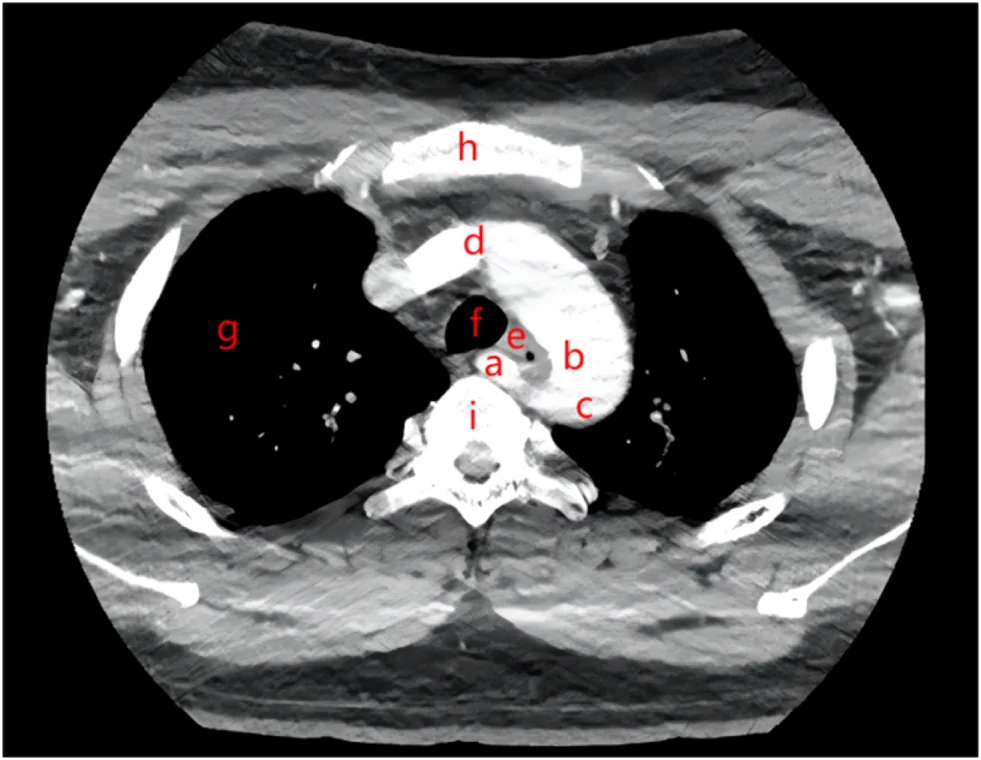
Preoperative contrast-enhanced CT of the neck demonstrates that the right subclavian artery courses behind the esophagus and is directly connected to the aortic arch. (A) Aberrant right subclavian artery; (B) Aortic arch; (C) Descending aorta; (D) Ascending aorta; (E) Esophagus; (F) Trachea; (G) Lung; (H) Sternum; (I) Vertebrae.

A 24-year-old male presented with a 1-month history of a right anterior neck mass. He had no hoarseness, dysphagia, or dyspnea. Physical examination revealed a firm, mobile, well-defined mass (5 × 5 × 4 cm) in the right thyroid lobe, with leftward tracheal deviation. Ultrasound on the same day showed a 53 × 50 × 40 mm isoechoic nodule with microcalcifications. Preoperative contrast-enhanced neck CT (18 October 2024) demonstrated a right thyroid lobe mass (51 × 38 × 52 mm) with heterogeneous enhancement, causing mild tracheal compression. Crucially, the right subclavian artery originated directly from the aortic arch, without a brachiocephalic trunk, and coursed posterior to the esophagus (Fig. 1), confirming ARSA and strongly suggesting a right NRLN.

**Figure 2.**
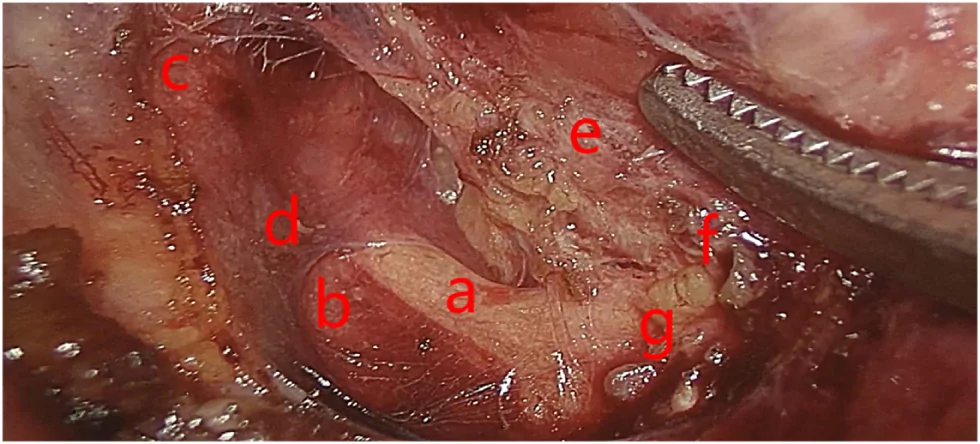
Intraoperative nerve exploration revealed that the main trunk of the cervical vagus nerve within the carotid sheath gives off a branch (the non-recurrent laryngeal nerve) that runs alongside the superior thyroid artery and directly traverses into the larynx. (A) Non-recurrent laryngeal nerve; (B) Main trunk of the cervical vagus nerve; (C) Common carotid artery; (D) Carotid sheath; (E) Thyroid capsule; (F) Superior thyroid artery; (G) Entry site of the non-recurrent laryngeal nerve into the larynx.

On 21 October 2024, the patient underwent endoscopic bilateral total thyroidectomy via a breast approach with central neck dissection. Intraoperative neural monitoring (IONM) was declined due to cost. During surgery, no RLN was found in the right tracheoesophageal groove or near the inferior thyroid artery. Instead, a nerve branch directly from the cervical vagus trunk within the carotid sheath was identified, running parallel to the superior thyroid artery and entering the larynx-consistent with Toniato Type I NRLN (Fig. 2). Meticulous dissection preserved the nerve and parathyroid glands. Intraoperative frozen section confirmed papillary thyroid carcinoma in the right lobe; the left lobe revealed nodular goiter. Total operative time was 100 minutes. No intraoperative or postoperative complications occurred (Clavien–Dindo grade 0). The patient was discharged on postoperative day 2 with normal vocal function. At 1, 2, 4, 7, and 14 months postoperatively, he remained asymptomatic with normal voice, and no recurrence was noted.

## Discussion

NRLN is a rare but clinically significant anomaly that increases the risk of nerve injury during thyroid surgery. A meta-analysis reported an NRLN injury rate of approximately 7%, nearly four times higher than that for the RLN^[^6^]^. Right NRLN is almost always associated with ARSA, which results from the absence of the brachiocephalic trunk and an abnormal origin of the right subclavian artery^[^7^]^. During normal growth and development, the right recurrent laryngeal nerve ascends to the larynx after passing around the subclavian artery^[^8^]^. However, in embryonic development, if the innominate artery (brachiocephalic trunk) is absent and the right subclavian artery originates abnormally, the vascular anomaly prevents the RLN from looping downward, causing the vagal branch to enter the larynx directly.

To systematically review the existing literature, we summarized representative published cases in Table 1. As shown, although the association between ARSA and NRLN has been well established, most studies employed the conventional open thyroidectomy approach, which entails significant trauma and a prolonged recovery period. Key features of this case include the following: (1) ETBA offered minimal invasiveness, faster recovery, and no cervical scarring; (2) despite declining IONM, preoperative contrast-enhanced CT revealed ARSA, enabling neural protection during surgery. To our knowledge, this is the first report of CT-predicted NRLN confirmed in endoscopic ETBA without IONM, underscoring the value of preoperative imaging.

**Table 1:** Typical cases where preoperative CT predicted the presence of an NPRLN and this was confirmed during thyroid surgery.

Author, year	Cases (*N*)	CT finding	NRLN type (Toniato)	Surgical approach	IONM used	Outcome
A N Hisham, 2001^[^9^]^	1	Without a vessel anomaly	Not reported	A standard Kocher collar incision (Open Thyroidectomy)	No	The NRLN was successfully identified and preserved, with normal vocal cord function postoperatively.
Bassam Abboud, 2007^[^10^]^	1	A retroesophageal ARSA	Not reported	A standard Kocher collar incision	No	Preserved normal vocal cord function
Diogo Casal, 2010^[^11^]^	1	Without a vessel anomaly	Not reported	A standard Kocher collar incision (Open Thyroidectomy)	No	The NRLN was successfully preserved, with no postoperative changes in voice quality.
Hong-Shik Choi, 2011^[^12^]^	2	Both cases underwent preoperative three-dimensional CTA. In the NRLN case: an ARSA was identified, which was associated with the NRLN; in the other case: a double aortic arch was observed, with normal course of the recurrent laryngeal nerve.	Not reported	A standard Kocher collar incision (Open Thyroidectomy)	No	Both cases successfully preserved the nerves without any alteration in voice quality.
B T Varghese, 2013^[^13^]^	1	Without a vessel anomaly	Not reported	A standard Kocher collar incision (Open Thyroidectomy)	No	The NRLN was successfully identified and preserved, with no postoperative complications occurring.
Rahim Mahmodlou, 2013^[^14^]^	1	No CT examination was performed preoperatively; postoperative CTA confirmed the presence of a retroesophageal ARSA.	Not reported	A standard Kocher collar incision (Open Thyroidectomy)	No	The NRLN was successfully identified and preserved; no postoperative vocal changes were observed, and no complications occurred.
Nazmi Yaşar Sayım, 2013^[^15^]^	2	Without a vessel anomaly	Both cases were Type 1.	A standard Kocher collar incision (Open Thyroidectomy)	No	Both cases exhibited no postoperative complications, with preserved neurological function.
C-C Wang, 2014^[^16^]^	1	A retroesophageal ARSA	Not reported	Robotic thyroidectomy	No	The NRLN was successfully preserved with no changes in sound.
James D Constable, 2017^[^17^]^	1	A retroesophageal ARSA (not described in the radiology report, but confirmed by the author upon reviewing the images)	Not reported	A standard Kocher collar incision (Open Thyroidectomy)	No	The lateral NRLN was successfully preserved without any complications.
Quang V Le, 2018^[^7^]^	4	All four patients were found to have an A retroesophageal ARSA on enhanced chest CT scans.	1 case of Type 1 and 3 cases of Type 2A	A standard Kocher collar incision (Open Thyroidectomy)	No	The NRLN were completely preserved in all four patients, with normal voice function and no complications observed.
Dimosthenis Chrysikos, 2019^[^18^]^	1	Without a vessel anomaly	Type 2A	A standard Kocher collar incision (Open Thyroidectomy)	No	The patient’s neurological function remained intact, and there was no change in her voice.
Tatsuya Furukawa, 2021^[^19^]^	1	A retroesophageal ARSA	Not reported	A standard Kocher collar incision (Open Thyroidectomy)	Yes	The NRLN remain intact, and vocal cord mobility is unaffected.
Feng Wen, 2021^[^20^]^	1	A retroesophageal ARSA and aberrant extracranial internal carotid artery	Not reported	A standard Kocher collar incision (Open Thyroidectomy)	No	No severe complications occurred, and the right NRLN was preserved (the left RLN was compromised due to tumor invasion; postoperative laryngoscopy revealed fixation of the left vocal cords). Postoperative hypocalcemia resolved after one week of calcium supplementation therapy, and hoarseness improved compared to preoperative levels.
Daqi Zhang, 2021^[^21^]^	2	A retroesophageal ARSA	One case each of Type 1 and Type 2	Robotic bilateral axillo-breast approach, BABA	Yes	In both cases, the NRLNs were identified, dissected, and preserved
Azza Mediouni, 2021^[^22^]^	2	Both cases exhibited a retroesophageal ARSA	Type 1 and Type 2A	A standard Kocher collar incision (Open Thyroidectomy)	No	Neither case exhibited postoperative vocal cord paralysis, and the NRLN was successfully preserved.
Ying Ki Lee, 2022^[^23^]^	1	A retroesophageal ARSA (MRI)	Not reported	A standard Kocher collar incision (Open Thyroidectomy)	Yes	The NRLN was successfully preserved, with excellent postoperative vocal function and no complications.
Manisha Dash, 2022^[^24^]^	1	Without a vessel anomaly	Not reported	A standard Kocher collar incision (Open Thyroidectomy)	Yes	The NRLN was successfully preserved without occurrence of vocal cord paralysis or hoarseness.
Birhanu Abdisa Tesso, 2023^[^25^]^	2	One case exhibited an A retroesophageal ARSA, while the other did not undergo CT examination.	Both cases were Type 2A.	A standard Kocher collar incision (Open Thyroidectomy)	No	Neither case exhibited neurological injury, and postoperative voice function remained normal.
Emin Gurleyik, 2023^[^26^]^	1	A retroesophageal ARSA	Not reported	A standard Kocher collar incision	Yes	The functional preservation of bilateral glottal movement controlled by the NRLN remains intact.
Ratan Medhi, 2024^[^3^]^	1	A retroesophageal ARSA	Type 2A	A standard Kocher collar incision (Open Thyroidectomy)	No	The NRLN was successfully preserved with normal bilateral vocal cord function and no postoperative complications.
Yumna Afzal, 2024^[^27^]^	1	A retroesophageal ARSA	Type 1	A standard Kocher collar incision	No	No post-operative complications,with intact voice and no dysphagia.
Hugo Pais Moreira, 2025^[^28^]^	1	A retroesophageal ARSA	Not reported	A standard Kocher collar incision	Yes	No vocal cord dysfunction or signs of nerve injury.
Present case, 2026	1	ARSA + absent innominate artery on CT	Type I	Endoscopic Thyroidectomy via Breast Approach (ETBA)	No	The NRLN was successfully preserved with no intraoperative or postoperative complications, and vocal cord function remained normal.

ARSA, aberrant right subclavian artery; IONM, intraoperative neuromonitoring; NRLN, non‑recurrent laryngeal nerve.

### Preoperative CT as a predictive tool

In a study of 1561 thyroid surgeries, CT achieved 100% sensitivity and specificity for detecting NRLN, versus 83.5% for ultrasound^[^29^]^. In our case, standard contrast-enhanced CT clearly depicted ARSA, enabling preoperative suspicion and intraoperative confirmation. Although CT angiography or MRI could provide additional detail, they were unnecessary here because the patient had no vascular compression symptoms. Moreover, even without IONM (declined by the patient), precise CT interpretation guided the surgeon to avoid the tracheoesophageal groove and directly identify the nerve arising from the vagal trunk, achieving successful preservation.

### NRLN classification and risk stratification

Currently, no universally accepted classification system exists for NRLN, and the Toniato classification is most widely used in clinical practice^[^25^]^. Type I NRLN originates directly from the cervical vagus trunk and descends into the larynx alongside the superior thyroid artery. Type IIa arises from the vagus nerve at the level of the inferior thyroid artery and runs parallel to it into the larynx. Type IIb also originates at the level of the inferior thyroid artery but enters the larynx after passing beneath or between branches of the inferior thyroid artery. According to the literature, Type I carries the highest risk of injury due to its intimate relationship with the superior thyroid vessels, followed by Type IIb, while Type IIa has the lowest risk^[^30,31^]^. Our case was a Type I NRLN. Although this subtype is most vulnerable to injury, preoperative contrast-enhanced CT successfully predicted its presence. During surgery, meticulous dissection ensured adequate nerve exposure, and extreme caution was exercised when ligating or transecting adjacent vessels, thereby effectively avoiding damage to the NRLN.

### Clinical signs warranting vigilance

Suspicion for an NRLN should be raised when imaging (CT, X-ray, or ultrasound) reveals an ARSA, or in rare conditions such as situs inversus or unexplained preoperative hoarseness or dysphagia associated with esophageal compression. Although these signs are not definitive, they should prompt heightened awareness and a modified dissection technique.

### Surgical strategy modification for suspected NRLN

Based on our experience, we recommend: (1) Do not routinely explore the tracheoesophageal groove; (2) Locate the cervical vagus and trace its branches; (3) If the nerve is not immediately seen, search near the cricothyroid joint and dissect retrograde; (4) Ligate superior vessels with great care; (5) Use fine, non-thermal instruments to avoid thermal injury.

### Limitations

This is a single case with a relatively short follow-up (14 months). IONM was not used, and specialized CTA was not performed. Nevertheless, we demonstrate that standard contrast-enhanced CT combined with meticulous technique can effectively predict and protect the NRLN, offering a practical approach in resource-limited settings or when patients decline monitoring.

## Conclusion

This case provides three key clinical takeaways: (1) Preoperative contrast-enhanced CT should routinely assess the aortic arch; if ARSA is identified, NRLN is highly probable. (2) When ARSA is present, prioritize identification of the cervical vagus trunk rather than the tracheoesophageal groove, and exercise extra care at the superior pole, especially for Toniato Type I. (3) IONM is not mandatory-safe nerve preservation can be achieved through thorough CT evaluation and precise anatomical dissection, which is a valuable alternative in settings without IONM or when patients refuse it.
